# Antibiotics Versus Natural Biomolecules: The Case of In Vitro Induced Bacteriospermia by *Enterococcus Faecalis* in Rabbit Semen

**DOI:** 10.3390/molecules24234329

**Published:** 2019-11-27

**Authors:** Michal Duracka, Norbert Lukac, Miroslava Kacaniova, Attila Kantor, Lukas Hleba, Lubomir Ondruska, Eva Tvrda

**Affiliations:** 1 Department of Animal Physiology, Faculty of Biotechnology and Food Sciences, Slovak University of Agriculture in Nitra, Tr. A. Hlinku 2, 949 76 Nitra, Slovakia; michaelduracka@gmail.com (M.D.); norbert.lukac@uniag.sk (N.L.); 2 Department of Fruit Growing, Viticulture and Enology, Faculty of Horticulture and Landscape Engineering, Slovak University of Agriculture in Nitra, Tr. A. Hlinku 2, 949 76 Nitra, Slovakia; kacaniova.miroslava@gmail.com; 3 Department of Bioenergy and Food Technology, Faculty of Biology and Agriculture, University of Rzeszow, Zelwerowicza St. 4, 35601 Rzeszow, Poland; 4 Department of Technology and Quality of Plant Products, Faculty of Biotechnology and Food Sciences, Slovak University of Agriculture in Nitra, Tr. A. Hlinku 2, 949 76 Nitra, Slovakia; kantor.spu@gmail.com; 5 Department of Microbiology, Faculty of Biotechnology and Food Sciences, Slovak University of Agriculture in Nitra, Tr. A. Hlinku 2, 949 76 Nitra, Slovakia; lukas.hleba@uniag.sk; 6 Institute of Small Farm Animals, Research Institute for Animal Production, Hlohovecká 2, 951 41 Lužianky, Nitra, Slovakia; ondruska@vuzv.sk

**Keywords:** *Enterococcus faecalis*, semen, antibiotics, antioxidants, oxidative stress, bacteriospermia, rabbits

## Abstract

Male subfertility is a global issue in human reproduction as well as in animal reproduction. Bacterial infection and semen contamination are still widely overlooked. As the collection of ejaculates is not a sterile process, it is necessary to add antimicrobial agents to avoid a possible depreciation of semen samples. As traditionally used antibiotics have been questioned because of an ever-increasing bacterial resistance, natural bioactive molecules could offer an alternative because of their antibacterial and antioxidant properties. As such, we decided to compare the effects of selected natural biomolecules (resveratrol-RES, quercetin-QUE and curcumin-CUR) with routinely used antibiotics in animal biotechnologies (penicillin-PEN, gentamicin-GEN and kanamycin-KAN) on the rabbit sperm vitality in the presence of *Enterococcus faecalis*. Changes in the sperm structural integrity and functional activity were monitored at 0, 2, 4 and 6 h. Computer-assisted sperm analysis (CASA) was used for the assessment of spermatozoa motility. Production of reactive oxygen species (ROS) was evaluated using chemiluminiscence, while the mitochondrial membrane potential (ΔΨm) was examined using the JC-1 dye. Finally, the sperm chromatin dispersion (SCD) test was used to assess DNA fragmentation, and changes to the membrane integrity were evaluated with the help of annexin V/propidium iodide. The motility assessment revealed a significant sperm motility preservation following treatment with GEN (*p* < 0.001), followed by PEN and CUR (*p* < 0.01). QUE was the most capable substance to scavenge excessive ROS (*p* < 0.001) and to maintain ΔΨm (*p* < 0.01). The SCD assay revealed that the presence of bacteria and antibiotics significantly (*p* < 0.05) increased the DNA fragmentation. On the other hand, all bioactive compounds readily preserved the DNA integrity (*p* < 0.05). In contrast to the antibiotics, the natural biomolecules significantly maintained the sperm membrane integrity (*p* < 0.05). The microbiological analysis showed that GEN (*p* < 0.001), KAN (*p* < 0.001), PEN (*p* < 0.01) and CUR (*p* < 0.01) exhibited the strongest antibacterial activity against *E. faecalis*. In conclusion, all selected biomolecules provided protection to rabbit spermatozoa against deleterious changes to their structure and function as a result of *Enterococcus faecalis* contamination. Therefore, administration of RES, QUE and/or CUR to rabbit semen extenders in combination with a carefully selected antibacterial substance may be desirable.

## 1. Introduction

Recently, one of the priorities of practical andrology is to determine the impact of urogenital infections on spermatozoa quality. Clinical studies are not fully in consensus about the effect of leukocytospermia and bacteriospermia on the function of male gametes, particularly in case of a contamination or colonization of ejaculates by bacteria [1,2]. The majority of urogenital infections remain asymptomatic [3], while damage to male reproductive cells highly depends on the type and concentration of bacteria present in ejaculates.

Bacteriospermia contributes to adhesion and agglutination of spermatozoa, leading to an abrupt decrease of motility. Bacteria can damage spermatozoa by a direct competition for nutrients or by the production of toxic metabolic intermediates or endotoxins [4,5].

The level of oxidative stress (OS) in ejaculates has been related to the presence of several infectious, contaminating or colonizing bacterial species. Bacteria have the ability to stimulate reactive oxygen species (ROS) generation via their toxic metabolites and virulent factors [6]. The role of ROS in cell biology is indubitable. In low amounts these molecules play physiologically important roles in sperm hyperactivation and acrosome reaction, whereas high ROS concentrations cause OS, endangering the fertilization potential of male gametes [7]. Mitochondria are the main source of intracellular ROS production in spermatozoa, and decide, if the cell remains viable or if it enters the apoptotic process [8]. OS as a result of ROS overproduction in mitochondria followed by lipid peroxidation culminates in DNA fragmentation. Cell death is furthermore caused by the presence of hydrogen peroxide, which readily diffuses through membranes [9,10,11].

**Figure 1:**
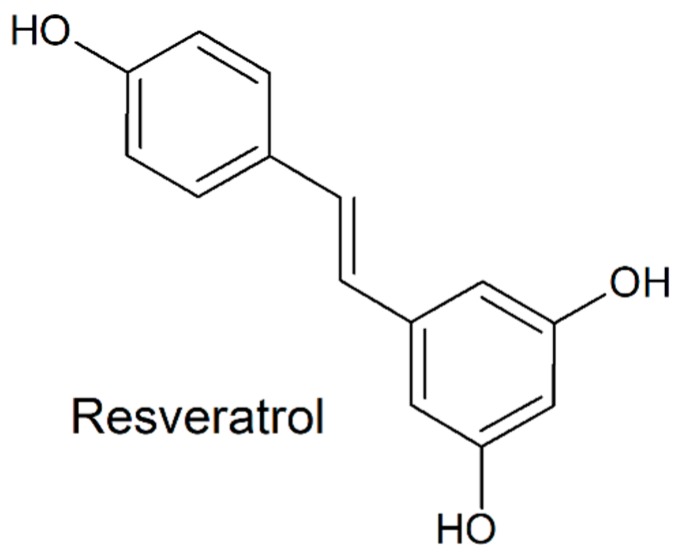
Chemical structure of resveratrol.

**Figure 2:**
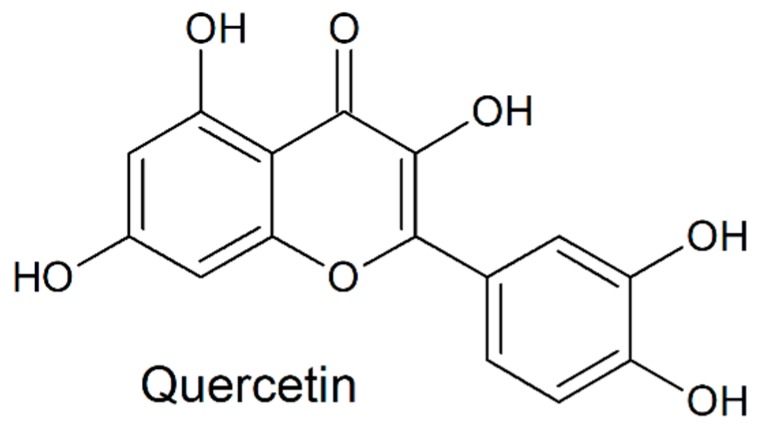
Chemical structure of quercetin.

**Figure 3:**
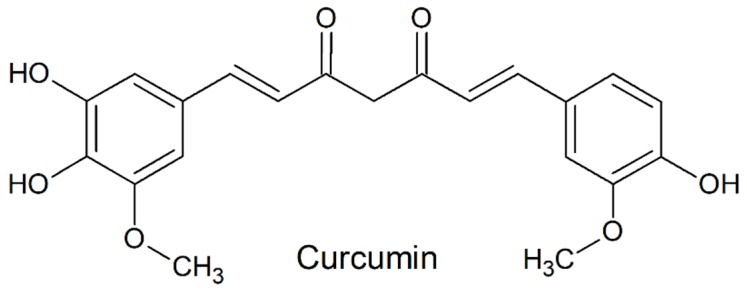
Chemical structure of curcumin.

While antibiotics (ATBs) are usually the first choice to counteract bacteriospermia, alternatives to these molecules could be beneficial in order to improve the resulting quality of stored spermatozoa, by decelerating the progress of antibiotic resistance or to avoid the potential toxic effects of ATBs on male gametes [12,13]. Three natural biologically active substances have recently attracted a widespread scientific attention. Resveratrol (RES; Figure 1) has been shown to be an efficient antimicrobial agent against several Gram-positive bacteria, including *E. faecalis* [13]. Its beneficial effects were also related to OS reduction in semen and sperm viability preservation [14]. A recent study revealed bacteriostatic effects of quercetin (QUE; Figure 2), which was stronger against Gram-positive bacteria in comparison to their Gram-negative counterparts [15]. Another study confirmed ROS-scavenging properties of QUE which may prevent spermatozoa alterations caused by OS [16]. Curcumin (CUR; Figure 3), found in turmeric, is well-known for to its antioxidant characteristics. Furthermore, its bactericidal activity was shown against Gram-positive as well as Gram-negative bacteria [17].

The aim of this study was a deep analysis of the efficiency of selected natural biomolecules (RES, QUE, CUR) and antibiotics traditionally used in animal biotechnologies (penicillin-PEN, gentamycin-GEN, kanamycin-KAN) [18,19,20] to decelerate the detrimental processes resulting from a co-culture of rabbit spermatozoa with an uropathogenic bacterium (*Enterococcus faecalis*) isolated from rabbit ejaculates. The methodic steps of this study were focused on a complex assessment of the essential components of the structural integrity and functional activity of male reproductive cells.

## 2. Results

### 2.1. In Vivo Experiments

Ejaculates were collected from 15 adult and healthy rabbit bucks with a sterile artificial vagina and subjected to microbiological analysis using the classical plate method and selective media. Nineteen bacterial colonies were isolated from the semen samples using the four-way streak plate method and identified by matrix-assisted laser desorption/ionization time-of-flight mass spectrometry (MALDI-TOF MS). The obtained data are summarized in Table 1.

### 2.2. In Vitro Experiments

Spermatozoa were obtained from rabbit ejaculates using density separation, and subjected to a co-culture with *E. faecalis*. The culture medium was furthermore supplemented with PEN (300 µg/mL), GEN (1000 µg/mL), KAN (and 80 µg/mL), RES (10 µmol/L), QUE (5 µmol/L) or CUR (1 µmol/L). At 0, 2, 4, and 6 h changes in the sperm structural integrity and functional activity were assessed using specific experimental procedures.

#### 2.2.1. Spermatozoa Motility

Initially, the motility assessment did not reveal any significant differences among the control or experimental groups. The first significant differences were observed after 2 h of incubation, when the motility in the positive control (PC), KAN and RES groups was significantly decreased (*p* < 0.05) when compared to the negative control (NC). The motility in the PEN, GEN and CUR groups significantly increased (*p* < 0.05) in comparison to PC.

Following 4 h the motility in the PC group substantially decreased (*p* < 0.01) in comparison to NC. In the meantime, the motility was significantly increased in GEN and CUR groups (*p* < 0.01) when compared to PC.

After 6 h the deteriorating effect of the bacterium was confirmed in the PC group as revealed by a significant decrease (*p* < 0.001) in comparison with NC. Among the selected antibiotics, motility was significantly retained (*p* < 0.001) only in the group treated with GEN when compared to PC. Among the selected bioactive molecules, CUR showed to be the most successful preserving agent of spermatozoa motility with a significant difference (*p* < 0.01) in the presence of *E. faecalis* (Table 2).

#### 2.2.2. Reactive Oxygen Species (ROS) Production

The motility decrease caused by the presence of *E. faecalis* was accompanied by an increased ROS generation, expressed as relative light units (RLU)/s/10^6^ sperm. Significant differences were observed already at the initial incubation time. With the increasing time of in vitro culture, higher ROS levels were recorded, particularly in the PC group (*p* < 0.001).

Administration of antibiotics led to a significant decrease of ROS (*p* < 0.001) when compared to PC. On the other hand, ROS levels were still significantly higher (*p* < 0.001) when compared to the NC group.

Experimental groups treated with RES or CUR did not exhibit any significant changes in the ROS levels when compared to NC, however in case of QUE, a significant rise of ROS production was recorded in comparison to NC (*p* < 0.05). Inversely, ROS levels were significantly decreased (*p* < 0.001) in each experimental group treated with natural biomolecules when compared to PC (Table 3).

#### 2.2.3. Mitochondrial Membrane Potential

The JC-1 assay revealed the first differences following exposure to the bacterium following 2 h of cultivation, as the mitochondrial membrane potential (ΔΨm) in the PC group was significantly decreased (*p* < 0.05) when compared to NC. The experimental group treated with QUE exhibited a significantly increased ΔΨm (*p* < 0.05), in comparison with PC.

After 4 h, a significant decrease of ΔΨm (*p* < 0.05) in the PC group was observed when compared to NC, however it remained significantly increased in the QUE and CUR groups (*p* < 0.05) when compared to PC. Following 6 h PC exhibited a significantly decreased ΔΨm (*p* < 0.01) when compared to NC. Groups supplemented with PEN and KAN also revealed significant deteriorations (*p* < 0.05) of ΔΨm when compared to NC. All selected bioactive molecules were able to preserve ΔΨm with a significant difference (*p* < 0.01) in comparison to PC (Table 4).

#### 2.2.4. DNA Fragmentation

The chromatin-dispersion test revealed differences in the sperm DNA integrity depending on the treatment.

First significant differences (*p* < 0.05) were noticed after 4 h in the PC group and groups treated with antibiotics, when compared to NC. The assessment following 6 h revealed a significant increase of DNA fragmentation in the PC, PEN, KAN and GEN groups (*p* < 0.05). On the other hand, bioactive molecules were able to more effectively maintain (*p* < 0.05) the DNA integrity in rabbit spermatozoa (Table 5) when compared to NC.

#### 2.2.5. Membrane Integrity

Table 6 displays the amount of spermatozoa with intact membranes (annexin-V and propidium iodide-PI negative cells) expressed as a percentage.

A significant decrease (*p* < 0.01) of membrane integrity was observed following 4 h in PC, when compared with NC. Assessment after 6 h showed a significant decrease of the membrane integrity (*p* < 0.001) in the PC group. Bioactive molecules exhibited significant positive effects on the membrane stability (*p* < 0.05) following comparison with the PC group.

Interesting data were collected in case of the assessment of PI-positive cells (Table 7). A significant increase (*p* < 0.05) of necrotic cells in PC could be observed already at initial time, when compared to NC. Differences between the PC and NC group became more pronounced and significant following 2 h of culture (*p* < 0.001). In case of the antibiotic-treated groups, the quantity of PI-positive cells was significantly (*p* < 0.05) increased when compared to NC. On the other hand, biomolecule-treated groups exhibited a decreased quantity of PI-positive cells (*p* < 0.05) when compared to PC.

#### 2.2.6. Microbiological Analysis

Table 8 reveals the antimicrobial effects of selected antibiotics and biomolecules on *E. faecalis* during in vitro induced bacteriospermia.

A significant decrease of bacterial growth was observed in the experimental groups supplemented with GEN (*p* < 0.001) and KAN (*p* < 0.01) in comparison with PC throughout the entire in vitro culture. Surprisingly, PEN was less effective in ceasing *E. faecalis* growth at selected timeframes of the experiment.

Among the bioactive molecules, CUR was the most effective in inhibiting the bacterial growth in comparison with PC (*p* < 0.01), nevertheless, it was less potent when compared to the antibiotics. In case of RES and QUE, no significant bacteriostatic effect was observed.

## 3. Discussion

During collection and/or sampling, microbial contamination of ejaculates is often observed, even if all the requirements for an aseptic and antiseptic handling of semen are met [21]. In order to avoid the loss of sperm vitality and the risk of an infection to females, readily available information about the bacterial species present in the ejaculate may be convenient for further semen processing and application. As opposed to traditional microbiological tools for the identification of bacteria, MALDI-TOF MS has emerged as a fast and straight-forward analytical technique to provide information on specific bacterial species present in complex biological samples [22]. Furthermore, based on the isolation, amplification and sequencing of DNA, Zampieri et al. [23] proved the accuracy and thus the suitability of the MALDI-TOF MS method in practical andrology. MALDI-TOF MS approach has already been successfully applied to study the bacterial profile of stallion [24], rooster [25] and bovine semen [23].

Our MALDI-TOF results indicate that rabbit semen may be heavily contaminated by diverse bacterial species, some of which are well-known uropathogens. Similarly to our findings, Mercier and Rideau [26] as well as Sinkovic [27] reported that the bacterial microflora in rabbit semen was primarily represented by *Enterobacteriaceae*, *Pseudomonas*, *Clostridium* and *Streptococcus*. At the same time, *Enterobacteriaceae* belonged to the predominant bacterial families detected in ram [28], bovine [23,29] and bubaline [29] semen.

The efficiency of sperm preparation methods to separate bacteria from the seminal fluid and to avoid possible negative effects of bacteriospermia on the sperm recovery rates are essential aspects of a successful sperm handling procedure [30]. The choice to process ejaculates prior to their use in artificial insemination (AI) or in vitro fertilization (IVF) is currently supported by studies in roosters [31] rabbits [31,32] and boars [31,33], where the semen samples often suffer from a significant contamination which may severely affect the final quality of the sample and subsequently the fertilization success. For this study, we selected the Percoll gradient separation. The suitability of this technique for rabbit semen has been confirmed in this study, as the initial motility of recovered spermatozoa varied from 70% to 80%. Furthermore, microbiological screening of the samples processed for the in vitro experiments did not detect any bacteria in the negative control, while only *E. faecalis* inocula were confirmed in the positive control and experimental groups. As such, we may suggest that the Percoll gradient separation may be a suitable technique to obtain a specimen of highly motile spermatozoa, yet clear from bacteria.

According to our data, *Enterococcus faecalis* was the most frequent bacterium found in 12 out of 15 samples used for the in vivo experiment. At the same time, the bacterium was reliably identified in 10 out of 19 bacterial colonies isolated from the semen samples. *E. faecalis* is present in normal intestinal flora, but it also has been identified also in both male and female urogenital tract. The Gram-positive bacterium causes mainly nosocomial infections as well as urinary tract infections, endocarditis and endophthalmitis [34,35]. What is more, *E. faecalis* has been repeatedly identified in semen cultures of infertile subjects and has been associated with a significantly poorer sperm quality along with changes in the composition of seminal plasma crucial for the sperm survival [3,36,37]. Based on the fact that *E. faecalis* was the predominant bacterium found in our samples combined with the emerging evidence on its detrimental effects on the sperm survival, we chose to observe its behavior in detail in our further in vitro experiments.

The effects of bacteria on the sperm function have long been underestimated, mainly due to only a short-term interaction between these two cell types during the ejaculation process. Despite such short interaction time in vivo, previous reports have strongly emphasized a significant relationship between a “silent ejaculate infection” and a decreased semen quality [1,38,39].

Sperm adhesion and agglutination during bacterial contamination leads to the loss of sperm motility and normal morphology [1,5,40]. In addition, deleterious effects of bacterial contamination may be explained by the expression of membrane factors, such as lipopolysaccharides, α- or β-haemolysins, cytotoxic necrotizing factors or the sperm immobilization factor [41,42]. Bacterial-mediated ROS production in semen is responsible for OS, which is currently accepted as one of the main factors leading to infertility [6]. A direct contact of bacterial toxins with spermatozoa may trigger apoptosis, which is considered to be an initial signal for sperm death [11,43].

As semen collection is not a completely sterile process, ATBs are commonly added to extenders used for AI or IVF in order to control possible microbial contamination during semen processing [12]. Since ATBs themselves may be toxic to spermatozoa [44,45,46], and because of an alarmingly increasing bacterial resistance [47], there is an urgent need to find alternatives to conventional ATBs to be used in animal reproduction biotechnologies [12]. Recent studies have emphasized a rebirth of natural bioactive molecules with a variety of beneficial properties, rich diversity, complexity and availability, lack of significant toxic effects and intrinsic biological activity [48,49].

The bioactive compounds used in this study were selected upon a strong body of evidence on their beneficial properties which could potentially provide a selective advantage to spermatozoa under a variety of stress conditions [14,16,50]. Furthermore, a number of studies reports on the ability of RES, QUE and CUR to exhibit in vitro antibacterial effects against *Enterococcus* or, at least, to counteract complications arising from its activity [51,52,53].

Our results show that among the selected biomolecules, GEN proved to be the best motion-preserving supplement. A similar observation was reported by Bresciani et al. [18] who developed a new extender for rabbit semen containing up to 40 mg/mL GEN. Nevertheless, we have to treat our results with caution as Jasko et al. [54] emphasized on the harmful effects of GEN on the motility of stallion spermatozoa at concentrations greater than 1 mg/mL. Parlevliet et al. [55] detected significant effects of GEN on hypermotility, which is a sign of capacitation and acrosome reaction. All these processes are interconnected, and their disruption may lead to a reduced sperm fertilization potential. Furthermore, it has been shown that GEN induces testicular OS by increasing ROS generation, followed by lipid and protein oxidation, double-strand DNA breaks and a decrease of the intricate antioxidant mechanisms [56,57].

On the other hand, it was shown that CUR could improve the sperm motility and overall viability of extended boar semen [58], as well as cryopreserved bovine [50] and rat spermatozoa [59], most likely through its potent antioxidant behavior. In the meantime, QUE was able to preserve the sperm vitality and functional activity of bovine spermatozoa exposed to induced OS [60] as well as of boar spermatozoa during the thawing and pre-insemination incubation process [61].

In this study, a significant ROS overgeneration was recorded in the positive control. As explained by Fraczek and Kurpisz [11], bacteria and their toxins may induce OS-inflicted damage to the reproductive cells, causing severe alterations to the sperm survival. Surprisingly, administration of ATBs did not lead to a desirable decrease of the amounts of ROS produced by damaged spermatozoa. Zhao and Drlica [62] demonstrated that aminoglycosides have the ability to induce OS, as GEN and KAN in particular have been associated with the most aggressive of free radicals—the hydroxyl radical. Therefore, it should be reconsidered to apply such ATBs to semen extenders or to suggest adequate antioxidant supplementation. In the meantime, we detected a notable potential of all natural biomolecules to scavenge excessive ROS, most likely as a result of the presence and/or activity of *E. faecalis*. Such behavior is corresponding to the conclusions of other studies reporting on a significant antioxidant effects of RES [14,60], QUE [16,61] and CUR [50,58,59] on male gametes under in vitro stress conditions.

Mitochondrial membrane potential is considered to be an important marker of structural, functional or oxidative damage in spermatozoa during bacterial infection. Increased ROS formation is strongly associated with the accumulation of JC-1 monomers in depolarized mitochondria [62]. Bacterial adhesion as well as soluble factors released by the bacteria may trigger rupture of the mitochondrial membrane [63]. Our JC-1 data particularly emphasize on the protective effects QUE may exhibit on the mitochondrial structures under bacteriospermia. As suggested by de Oliveira et al. [64] and Tvrda et al. [16] the primary intercellular target of QUE could be mitochondria. In addition to its antioxidant properties, the molecule provides protection and regulation of critical mitochondrial processes, including the electron transport chain and oxidative phosporylation, that could affect the metabolism and behavior of male gametes.

The majority of reports focused on bacteriospermia emphasizes on elevated sperm DNA fragmentation in contaminated ejaculates [65,66]. Accordingly, our study has clearly confirmed an increased sperm DNA damage as a result of in vitro induced semen infection by *E. faecalis*. DNA integrity has become a crucial parameter in the evaluation of male fertilization potential. Moskovtsev et al. [67] reported that 48% of the subjects suffering from bacteriospermia (particularly in case of the *Enterococci* species) had high sperm DNA damage (≥30%)—more than patients suffering from varicocele (30%) or idiopathic infertility (22%). According to Tvrdá et al. [68] the presence of potentially uropathogenic bacteria in semen samples collected from clinically healthy bulls was associated with a high DNA fragmentation index.

The use of ATBs in modern biotechnologies carries the risk of significant side effects. In our study, exposure to ATBs led to increased sperm DNA fragmentation when compared to the groups containing bioactive molecules. Recent studies emphasize on the deleterious effects of bactericidal ATBs on the DNA stability of mammalian spermatozoa [44,45,46,57,69,70]. On the other hand, various studies, including ours, pointed out the DNA-protective properties of RES, QUE and CUR, which were capable of preserving the male fertility potential under a variety of stress conditions [14,16,50,58,59].

The increased percentage of apoptotic sperm in contaminated ejaculates could be attributed to actual bacteria or leukocytes. Bacterial endotoxins are known to trigger the expression of Toll-like receptors 2 and 4 in the sperm membranes [71,72]. On the other hand, aminoglycoside ATBs are able to induce programmed cell death through ROS overproduction, lipid peroxidation, cytochrome c leakage and caspase activation [73,74,75]. Based on this knowledge, we may explain the highest production of ROS detected in the groups containing KAN and GEN. Inversely, Aktas et al. [76] revealed that CUR could protect testicular cells from heavy metal-induced apoptosis. Previous studies suggest that the suppression of the ROS-activated Jun N-terminal kinase pathway is associated with the anti-apoptotic effect of QUE [77]. Moreover, anti-apoptotic properties of RES were confirmed through the extracellular signal-regulated kinase pathway [78]. Based on these findings, we may explain an increased percentage of spermatozoa with intact membranes observed in groups treated with selected antioxidants.

To our knowledge, this study is the first experimental simulation of an in vitro bacteriospermia in animal ejaculates. In vitro bacterial infection of human ejaculates was introduced by Fraczek et al. [79]. Similarly to our report, the authors observed a significantly decreased sperm motility accompanied by an increased proportion of necrotic cells. Bacteriospermia is accompanied by cell death through several distinct modalities, including necrosis. It is generally acknowledged that necrosis is triggered by ROS production, ATP depletion and TNF-α synthesis that are induced upon bacterial infection [80]. Interestingly, ATBs were not fully able to protect cells against necrosis. In this sense it is of relevance to note that earlier studies testing the effect of aminoglycosides on mammalian cells have reported a higher occurrence of necrotic processes, such as endonuclease G translocation, activation of calpain and cathepsin D [81,82]. Thus, it may be plausible that the presence of ATBs may cause multiple forms of cell death depending on the concentration and duration of treatment [83]. On the other hand, the selected antioxidants used in our study were able to decrease the ratio of necrotic cells. RES, QUE as well as CUR can easily permeate through the cytoplasm and accumulate in membranous structures [84], and exhibit anti-inflammatory effects mediated by TNF-α inhibition [85] and ROS scavenging [14,15].

The microbiological assessment of the control and experimental samples revealed that all ATBs performed better against the growth of *E. faecalis* in comparison with the selected natural biomolecules. As expected, and indeed indicated in previous reports, GEN and KAN are considered to be antibacterial substances of choice for semen extenders in rabbits [18,19], stallions [86] and bulls [87]. Nevertheless, the ever-increasing bacterial tolerance and/or resistance towards antibiotics needs to be remembered. Bennemann et al. [88] concluded that bacteria found in 86% of semen samples collected from boars, showed an important resistance to PEN (>75%) and lincomycin (>87.5%). What is more, Machen et al. [89] studied the time trends for bacterial species and resistance patterns in human semen. *Streptococcus*, *Staphylococcus* and *Enterococcus* were the most prevalent. Although the resistance data for the pathogens showed minimal statistically significant difference in a 5-year interval, the selected species did show a trend towards an increasing resistance particularly against PEN, ampicillin/sulbactam and erythromycin. Jayarao and Oliver [90] studied the resistance of *Streptococcus* and *Enterococcus* species isolated from bovine secretions. Their experiments revealed that amongst the most sensitive antibiotics streptomycin (85.4%) was most prevalent, followed by KAN (19%) and GEN (2.2%). Inversely, Bresciani et al. [91] observed GEN resistance of numerous bacterial isolates collected from boar semen. All in all, it may be suggested that ATBs currently used in sperm cell preparations may need to be varied in order to avoid future complications resulting from bacterial resistance.

While it has been suggested that the natural biomolecules used in our experiments exhibit antibacterial effects against *E. faecalis*, only CUR was able to cease the bacterial growth in the experimental groups. Various explanations have been provided with respect to the antibacterial mechanism of action of CUR. Rai et al. [92] reported that CUR interacts with the assembly of bacterial protofilaments, leading to an inhibition of bacterial cytokinesis and cell proliferation. Furthermore, it has been revealed that CUR binds to peptidoglycan and perturbs the bacterial membrane integrity, leading to cell lysis. What is more, CUR also exerts a substantial antibacterial activity when used at a subinhibitory dose in combination with various ATBs [93] and increases the bacterial sensitivity towards β-lactam antibiotics such as PEN and methicillin [94].

The relatively low bactericidal activity of RES, QUE and CUR may lie in the concentrations used for our experiments. Reports suggesting significant antibacterial properties of all biomolecules [13,15,17,95] had been working with concentrations well above 1 mmol/L, whereas our study utilizes concentrations of 10 µmol/L RES, 5 µmol/L QUE and 1 µmol/L CUR. In our case, the in vitro co-culture includes two significantly different types of cells. As our previous standardization study indicates, RES, QUE as well as CUR act in an interesting dichotomy: low concentrations exhibit beneficial effects of the sperm survival, whereas their high concentrations are cytostatic [96]. More significant antibacterial properties of RES, QUE and CUR could have been obtained when using higher concentrations, nevertheless the sperm survival would have been endangered. Because none of the biomolecules was able to completely reverse bacterial contamination when compared to the performance of the antibiotics, we may hypothesize that their beneficial effects on the sperm structural integrity and functional activity might have been associated with their ability to counteract ROS and endotoxins released into the culture as a result of the presence of *E. faecalis*.

## 4. Materials and Methods

### 4.1. Animals, Sample Collection and Processing

Fifteen male rabbits (New Zealand white broiler line) were used in this experiment. The animals were 4 months old, with a weight of 4.0 ± 0.2 kg and kept at the experimental farm of the Animal Production Research Centre Nitra, Slovakia. The rabbits were housed in a partially air-conditioned rabbit house under a photoperiod of 16L:8D, kept in individual cages and fed with a commercial diet. Water was provided ad libitum. Air temperature of 20–24 °C and relative humidity of 65% were maintained in the rabbit house. Institutional and national guidelines on the care and use of animals were followed, and all the experimental procedures were approved by the State Veterinary and Food Institute of Slovak Republic (no. 3398/11- 221/3) and Ethics Committee.

One ejaculate was obtained from each rabbit using an artificial vagina. In order to avoid possible contamination, sterile collection tubes were used, and the vagina was washed with soap and rinsed with boiling water following each sample collection. Prior to ejaculation the animals were allowed to urinate, and subsequently their genitals were washed with soapy water. Single-use gloves were changed between every collection.

Immediately upon collection, the sperm concentration and motility were assessed in each ejaculate. Only samples with a minimum 70% motility (>5 µm/s) were accepted for subsequent experiments.

One hundred µL of each sample was transferred into the MacConkey agar (Biomark, Pune), blood agar (Himedia Laboratories Pvt. Ltd., India), tryptone soya agar (Himedia Laboratories) and MRS agar (Biolife, Italy) and subsequently incubated at 37 °C for 48–72 h. The microorganisms were purified by the four-way streak plate method [97].

### 4.2. MALDI-TOF MS

Microbiological analysis of samples was carried out by the matrix-assisted laser desorption/ionization time-of-flight mass spectrometry (MALDI-TOF MS; Bruker Daltonics, Bremen, Germany) based on protein fingerprints. Single colonies of fresh overnight cultures were used for the ethanol-formic acid extraction. Each sample spot was covered with 2 µL of matrix solution (saturated solution of α-cyano-4-hydroxycinnamic acid in 50% acetonitrile with 2.5% trifluoroacetic acid; Bruker Daltonics) and air-dried for 15 min. Raw data of protein spectrum of each isolate was imported into the Biotyper software, version 2.0 (Bruker Daltonics) and analyzed [97].

### 4.3. In Vitro Experimental Design

The liquid cultivation medium for in vitro experiments contained phosphate-buffered saline (Dulbecco’s PBS without calcium and magnesium; Sigma-Aldrich, St. Louis, MO, USA), 10% bovine serum albumin (BSA; Sigma-Aldrich) and 5% glucose (Sigma-Aldrich). The isolated bacterial culture was resuspended in the medium and cultured for 24–48 h at 36 °C. The final concentration of the *E. faecalis* bacterial culture was adjusted to 0.3 McF (0.9 × 10^8^ CFU/mL) using a densitometer (DEN–1 McFarland Densitometer; Grant-bio, Cambridge, UK).

Rabbit ejaculates for the in vitro experiments were collected from the same animals and under the same conditions as for the bacterial identification. To obtain an adequate sample volume for the subsequent experiments, heterospermia was prepared by pooling at least 7 rabbit ejaculates with a minimum 70% initial motility.

In order to purify spermatozoa from somatic cells, bacteria, immature spermatids and diploid spermatocytes, rabbit ejaculates were run through a density separation using Percoll Plus^®^ (Merck, Darmstadt, Germany). A volume of 400 μL of semen diluted in 1 mL PBS was subjected to centrifugation (5 min, 3000 RPM). The supernatant was discarded, and the pellet was resuspended in 1 mL PBS. One mL of the mixture was added to 3 mL of a pre-warmed (37 °C) Percoll gradient (1.5 mL, 90% and 1.5 mL, 45%), and subsequently centrifuged (30 min, 1600 RPM). The bottom layer was transferred to an Eppendorf tube and centrifuged (5 min, 3000 RPM). Viable spermatozoa located on the top layer were transferred to a clean microtube, washed twice with PBS and resuspended in the liquid culture medium.

Because of a varying biological activity of conventional ATBs, and taking into account their potential toxic effects on the bacterial or eukaryotic cell, the concentrations of 300 µg/mL PEN, 1000 µg/mL GEN and 80 µg/mL KAN were selected based on the European Council Directive 90/429/EEC, Annex C2 [20] as well as information gathered from previous scientific reports focused on the preservation possibilities of rabbit ejaculates [18,19,20]. The final doses of ATBs were furthermore validated in our laboratory settings prior to the in vitro experiments. Specific concentrations of bioactive molecules for the in vitro cultures—10 µmol/L RES, 5 µmol/L QUE and 1 µmol/L CUR were chosen upon our previous investigation on their beneficial and/or harmful effects on the rabbit sperm behavior [96], revealing an optimal concentration pattern unique to each biomolecule in ensuring the highest sperm survival. All selected supplements were purchased from Sigma-Aldrich. Antibiotics as well as bioactive molecules were diluted in fresh culture medium.

Eight groups were prepared for the subsequent experiments—two controls and six experimental groups. The negative control (NC) consisted of the culture medium and isolated spermatozoa, while the positive control (PC) contained the sperm sample and the isolated culture of *E. faecalis*, the most prevalent bacterium identified in rabbit ejaculates. The experimental groups consisted of the culture medium, spermatozoa, *E. faecalis* and a selected antibiotic or biomolecule as shown in Table 9. All in vitro experiments were performed in quintiplicates.

The samples were cultured at 37 °C and at 0, 2, 4, and 6 h possible changes in the structural integrity and functional activity of male reproductive cells were assessed using specific techniques.

### 4.4. Computer-Assisted Semen Analysis

Spermatozoa motility (MOT; percentage of motile spermatozoa; motility > 5 μm/s; %) was evaluated using computer-assisted sperm analysis (CASA, Version 14.0 TOX IVOS II.; Hamilton-Thorne Biosciences, Beverly, MA, USA). Ten µL of each sample were applied to the Makler counting chamber (depth 10 µm; Sefi Medical Instruments, Haifa, Izrael), placed to a pre-warmed plate and introduced to the machine. Ten microscopic fields were subjected to each analysis in order to include at least 300 cells [96].

### 4.5. Reactive Oxygen Species (ROS) Production

ROS generation in the samples was analyzed by the chemiluminescence assay using luminol (5-amino-2,3-dihydrophthalazine-1,4-dione; Sigma-Aldrich) as the probe. The blank consisted of 100 µL of PBS (Sigma-Aldrich), while the tested samples consisted of luminol (2.5 µL, 5 mM) and 100 µL of control (in triplicates) or experimental sample. The negative control consisted of PBS and luminol, whereas the positive control contained PBS, luminol and 12.5 µL 30% hydrogen peroxide (8.8 M; Sigma-Aldrich). Chemiluminiscence was recorded using a combined spectro-fluoro-luminometer (Glomax Multi+, Promega, Madison, WI, USA) for 15 min [16].

### 4.6. Assessment of the Mitochondrial Membrane Potential

Mitochondrial membrane potential (ΔΨm) was assessed using the Mitochondrial Membrane Potential Assay Kit (Cayman Chemical, Ann Arbour, MI, USA). The cytofluorimetric cationic dye JC-1 enters to the mitochondria and subsequently changes its fluorescent characteristics based on its aggregation activity. In healthy cells with a high ΔΨm, JC-1 forms complexes, emitting a red fluorescent light. However, in cells with a low ΔΨm the JC-1 dye remains as a monomer, followed by the emission of a green fluorescent light. The analysis was performed using the Glomax Multi+ combined spectro-fluoro-luminometer (Promega) on dark 96-wells plates. The resulting ΔΨm was expressed as the rate of JC-1 complexes to JC-1 monomers [68].

### 4.7. The Sperm Chromatin Dispersion Test

DNA fragmentation was measured using the commercially available kit Halomax (Halotech, Madrid, Spain) optimized for rabbit spermatozoa. The cells were fixed in an agarose matrix on a microscope slide, subsequently DNA was denatured, and the nuclear proteins were removed. Following exposure to SYBR Green (Sigma-Aldrich) and fluorescent visualization using a fluorescent microscope (Leica DMI6000 B; Wetzlar, Germany), the result was determined observing the presence/absence of the “halo effect” equivalent to fragmented/non-fragmented DNA. At least 300 cells were included in each analysis [65].

### 4.8. Sperm Membrane Integrity

To detect male gametes with a compromised membrane integrity, annexin-V-FLUOS kit (Roche Applied Sciences, Basel, Switzerland) was used. In apoptotic cells the phospholipid-phosphatidylserine was translocated from the internal to the external side of the plasmatic membrane. To differentiate apoptosis from necrosis, propidium iodide (PI) was used, as this dye does not pass through the intact plasmatic membrane, however, it binds specifically to DNA. The evaluation was realized on the Leica DMI6000 B fluorescent microscope with appropriate filters for green, red and blue fluorescence, and at least 300 cells were counted in each group:

• Blue fluorescence—total count of cells (DAPI counterstain; 4′,6-diamidino-2-phenylindole; Sigma-Aldrich);
• Green fluorescence—apoptotic cells (annexin-V);
• Red fluorescence—necrotic cells (PI) [98].

### 4.9. Microbiological Analysis

Each control and experimental group was subjected to a repeated microbiological analysis based on the aforementioned cultivation using specific agars, incubation periods, purification methods and MALDI-TOF in order to verify the success of the sperm purification (absence of any bacteria in the negative control) and to confirm the sole presence of *E. faecalis* in the positive control and experimental groups.

If case any other bacteria were present in the tested groups, these were to be excluded from the final statistical analysis of collected experimental data.

Positive control and experimental samples that tested positive for the presence of *E. faecalis* exclusively, were furthermore cultured at the *Enterococcus* selective agar (Sigma-Aldrich) at 37 °C for 48–72 h and the plate dilution method was used to quantify the CFU/mL counts of *E. faecalis* following exposure to selected concentration of antibiotics or biomolecules [99].

### 4.10. Statistical Analysis

All data were subjected to statistical analysis using the GraphPad Prism program (version 6.0 for Windows, Graphpad Software incorporated, San Diego, California, USA, http://www.graphpad.com/). Results are quoted as arithmetic mean ± standard error of mean (SEM). Differences between control and experimental groups were statistically evaluated by one-way ANOVA with Sidak’s multiple comparison test. The positive control was compared to the negative control exclusively. All experimental groups were compared with both controls. The level of significance for the comparative analysis was set at * *p* < 0.05; ** *p* < 0.01; *** *p* < 0.001.

## 5. Conclusions

Because of the constantly increasing bacterial resistance to traditionally used antibiotics in semen extenders, their use in practical andrology needs to be treated with caution. Furthermore, it is necessary to seek new supplements that could either effectively suppress bacterial colonization or provide a certain advantage to male gametes, thereby prolonging their viability.

The data gathered from our experiments suggest that while penicillin, gentamicin and kanamycin were more effective in the preservation of the sperm motility; resveratrol, quercetin and curcumin provided greater protection to the mitochondrial function, membrane and DNA integrity in comparison to traditional antibiotics. What is more, all natural biomolecules behaved as outstanding antioxidants, decreasing the risk of oxidative insults to male gametes as a possible result of bacteriospermia. Nevertheless, our microbiological analysis revealed that conventional antibiotics exhibited a stronger antibacterial activity against *E. faecalis* when compared to the natural biomolecules.

Summarizing the results of our analyses we may conclude that although curcumin, quercetin and resveratrol do not exhibit a significant bactericidal activity, all of them act as protective agents counteracting the deleterious changes to the sperm structure and function as a result of bacteriospermia. It seems that all beneficial properties of the selected bioactive molecules in this study are related to their radical-scavenging properties, subsequently decreasing the risk for the sperm membrane and DNA damage.

Nevertheless, the potential implementation of curcumin, quercetin or resveratrol as supplements for rabbit semen extenders must rely on follow-up studies that should investigate the synergy of biologically active substances with different activity profiles, especially antibiotics and natural biomolecules mutually. At the same time, further experiments shall be required to assess the effectiveness of selected biomolecules against other bacterial species commonly found in rabbit ejaculates.

## Figures and Tables

**Table 1:** Bacterial strains isolated from rabbit ejaculates.

Sample			Log Score
1	+	*Pseudomonas oryzihabitans*	1.85
2	+++	*Acinetobacter baumannii*	2.375
3	+++	*Acinetobacter baumannii*	2.388
4	+	*Pseudomonas sp.*	1.728
5	+	*Pseudomonas oryzihabitans*	1.973
6	+	*Pseudomonas oryzihabitans*	1.71
7	+++	*Enterococcus faecalis*	2.377
8	++	*Acinetobacter baumannii*	2.25
9	+++	*Acinetobacter baumannii*	2.46
10	+++	*Acinetobacter baumannii*	2.406
11	+++	*Enterococcus faecalis*	2.441
12	+++	*Enterococcus faecalis*	2.436
13	+++	*Enterococcus faecalis*	2.485
14	+++	*Enterococcus faecalis*	2.46
15	+++	*Enterococcus faecalis*	2.481
16	+++	*Enterococcus faecalis*	2.495
17	+++	*Enterococcus faecalis*	2.468
18	+++	*Enterococcus faecalis*	2.459
19	+++	*Enterococcus faecalis*	2.442

Legend: +++ highly probable species identification (log score ≥2.30); ++ highly probable identification of the genus and probable identification of the species (log score: 2.00–2.29); + reliable identification of the genus (log score: 1.70–1.99).

**Table 2:** The effect of selected antibiotics and bioactive molecules on the spermatozoa motility (MOT) during induced bacteriospermia.

MOT [%]	0 h	2 h	4 h	6 h
NC	70.00 ± 5.39	62.00 ± 5.44	53.67 ± 4.04	42.67 ± 5.65
PC	68.00 ± 4.70	32.67 ± 3.18 ^*NC^	9.00 ± 3.51 ^**NC^	0.33 ± 0.03 ^***NC^
PEN	69.00 ± 5.10	53.00 ± 4.20 ^*PC^	36.67 ± 6.67 ^*PC^	28.67 ± 2.39 ^**PC^
GEN	70.33 ± 2.80	58.00 ± 4.70 ^*PC^	50.00 ± 5.90 ^**PC^	34.00 ± 4.26 ^***PC^
KAN	69.33 ± 6.84	45.67 ± 6.33 ^*NC^	29.67 ± 5.20 ^*NC, *PC^	20.00 ± 1.15 ^*NC, *PC^
RES	68.00 ± 5.39	41.67 ± 3.83 ^*NC^	26.33 ± 6.19 ^*NC, *PC^	15.67 ± 0.67 ^**NC, *PC^
QUE	69.67 ± 4.06	49.67 ± 6.79	36.67 ± 5.29 ^*PC^	22.00 ± 1.52 ^*PC^
CUR	69.33 ± 4.37	55.33 ± 5.28 ^*PC^	44.00 ± 2.26 ^**PC^	26.33 ± 2.39 ^**PC^

Legend: * *p* < 0.05; ** *p* < 0.01; *** *p* < 0.001; NC—negative control; PC—positive control; PEN—penicillin; GEN—gentamicin; KAN—kanamycin; RES—resveratrol; QUE—quercetin; CUR—curcumin; ^NC^—compared to the negative control; ^PC^—compared to the positive control.

**Table 3:** The effect of selected antibiotics and bioactive molecules on reactive oxygen species (ROS) generation during induced bacteriospermia.

ROS [RLU/s/10^6^ Sperm]	0 h	2 h	4 h	6 h
NC	1.54 ± 0.26	5.44 ± 0.60	8.21 ± 0.33	10.86 ± 0.34
PC	5.43 ± 0.35 ^***NC^	11.51 ± 0.25 ^***NC^	17.98 ± 1.10 ^***NC^	26.85 ± 0.31 ^***NC^
PEN	4.29 ± 0.26 ^***NC^	9.23 ± 0.10 ^***NC, **PC^	14.45 ± 1.29 ^**NC^	17.93 ± 0.90 ^***NC, ***PC^
GEN	4.11 ± 0.21 ^***NC, *PC^	9.15 ± 0.54 ^***NC, *PC^	14.71 ± 0.92 ^***NC^	18.06 ± 1.68 ^***NC, ***PC^
KAN	4.42 ± 0.30 ^***NC^	8.83 ± 0.46 ^***NC, **PC^	14.46 ± 0.98 ^**NC^	18.58 ± 1.01 ^***NC, ***PC^
RES	2.87 ± 0.14 ^*NC, ***PC^	7.39 ± 0.15 ^*NC, ***PC^	11.38 ± 0.50 ^***PC^	14.48 ± 0.71 ^***PC^
QUE	2.91 ± 0.14 ^*NC, ***PC^	7.19 ± 0.15 ^***PC^	11.86 ± 0.55 ^**PC^	15.21 ± 0.48 ^*NC, ***PC^
CUR	2.45 ± 0.17 ^***PC^	6.89 ± 0.24 ^***PC^	11.08 ± 0.37 ^***PC^	13.90 ± 0.70 ^***PC^

Legend: * *p* < 0.05; ** *p* < 0.01; *** *p* < 0.001; NC—negative control; PC—positive control; PEN—penicillin; GEN—gentamicin; KAN—kanamycin; RES—resveratrol; QUE—quercetin; CUR—curcumin; ^NC^—compared to the negative control; ^PC^—compared to the positive control.

**Table 4:** The effect of selected antibiotics and bioactive molecules on the ΔΨm during induced bacteriospermia.

JC-1 [Units]	0 h	2 h	4 h	6 h
NC	0.48 ± 0.14	0.36 ± 0.10	0.25 ± 0.05	0.18 ± 0.02
PC	0.36 ± 0.11	0.24 ± 0.10 ^*NC^	0.15 ± 0.07 ^*NC^	0.09 ± 0.02 ^**NC^
PEN	0.41 ± 0.13	0.28 ± 0.10	0.21 ± 0.04	0.15 ± 0.02 ^*NC^
GEN	0.39 ± 0.12	0.27 ± 0.10	0.21 ± 0.03	0.13 ± 0.04
KAN	0.40 ± 0.13	0.28 ± 0.12	0.20 ± 0.04	0.14 ± 0.02 ^*NC^
RES	0.45 ± 0.14	0.32 ± 0.10	0.22 ± 0.04	0.17 ± 0.03 ^**PC^
QUE	0.46 ± 0.12	0.35 ± 0.11 ^*PC^	0.25 ± 0.05 ^*PC^	0.18 ± 0.03 ^**PC^
CUR	0.45 ± 0.12	0.31 ± 0.11	0.23 ± 0.03 ^*PC^	0.17 ± 0.03 ^**PC^

Legend: * *p* < 0.05; ** *p* < 0.01; *** *p* < 0.001; NC—negative control; PC—positive control; PEN—penicillin; GEN—gentamicin; KAN—kanamycin; RES—resveratrol; QUE—quercetin; CUR—curcumin; ^NC^—compared to the negative control; ^PC^—compared to the positive control.

**Table 5:** The effect of selected antibiotics and bioactive molecules on the sperm DNA fragmentation index (FI) during induced bacteriospermia.

DNA FI [%]	0 h	2 h	4 h	6 h
NC	3.42 ± 0.26	5.87 ± 0.66	7.56 ± 0.87	10.63 ± 1.01
PC	4.37 ± 0.21	8.06 ± 0.52	12.29 ± 0.87 ^*NC^	17.93 ± 1.23 ^*NC^
PEN	4.71 ± 0.33	8.69 ± 0.47	12.90 ± 0.90 ^*NC^	17.66 ± 1.34 ^*NC^
GEN	4.74 ± 0.25	8.66 ± 0.39	12.77 ± 0.78 ^*NC^	17.10 ± 1.52 ^*NC^
KAN	4.72 ± 0.33	8.72 ± 0.41	13.32 ± 0.88 ^*NC^	17.17 ± 2.01 ^*NC^
RES	3.86 ± 0.31	6.99 ± 0.50	8.55 ± 0.67	12.25 ± 1.05 ^*PC^
QUE	3.73 ± 0.17	6.91 ± 0.43	8.90 ± 0.77	12.34 ± 1.03 ^*PC^
CUR	3.91 ± 0.36	6.83 ± 0.34	8.33 ± 0.92	11.94 ± 1.02 ^*PC^

Legend: * *p* < 0.05; ** *p* < 0.01; *** *p* < 0.001; NC—negative control; PC—positive control; PEN—penicillin; GEN—gentamicin; KAN—kanamycin; RES—resveratrol; QUE—quercetin; CUR—curcumin; ^NC^—compared to the negative control; ^PC^—compared to the positive control.

**Table 6:** The effect of selected antibiotics and bioactive molecules on membrane integrity during induced bacteriospermia.

A^−^/PI^−^ [%]	0 h	2 h	4 h	6 h
NC	92.59 ± 2.17	90.27 ± 3.44	85.91 ± 4.53	82.09 ± 2.78
PC	91.52 ± 3.56	84.20 ± 3.22	75.91 ± 4.92 ^**NC^	68.95 ± 3.25 ^***NC^
PEN	92.05 ± 2.02	87.09 ± 3.24	80.15 ± 3.83	76.52 ± 3.01
GEN	91.91 ± 1.53	87.10 ± 2.71	80.25 ± 4.20	76.26 ± 2.29
KAN	91.94 ± 1.65	87.66 ± 3.54	80.13 ± 3.78	75.63 ± 2.51
RES	92.98 ± 2.34	88.89 ± 3.07	81.51 ± 3.59	77.86 ± 2.98 ^*PC^
QUE	93.22 ± 2.35	88.75 ± 2.34	81.77 ± 3.06	77.48 ± 3.32 ^*PC^
CUR	93.52 ± 1.58	88.55 ± 2.35	81.66 ± 3.31	77.99 ± 3.75 ^*PC^

Legend: * *p* < 0.05; ** *p* < 0.01; *** *p* < 0.001; NC—negative control; PC—positive control; PEN—penicillin; GEN—gentamicin; KAN—kanamycin; RES—resveratrol; QUE—quercetin; CUR—curcumin; ^NC^—compared to the negative control; ^PC^—compared to the positive control.

**Table 7:** The effect of selected antibiotics and bioactive molecules on cell necrosis in induced bacteriospermia.

PI^+^ [%]	0 h	2 h	4 h	6 h
NC	2.55 ± 0.28	3.82 ± 0.26	5.59 ± 0.69	7.74 ± 0.61
PC	5.53 ± 0.72 ^*NC^	12.32 ± 1.11 ^***NC^	18.30 ± 1.74 ^***NC^	25.09 ± 2.04 ^***NC^
PEN	3.97 ± 0.58	9.79 ± 1.82 ^*NC^	14.66 ± 1.57 ^*NC^	18.53 ± 2.74 ^*NC^
GEN	4.58 ± 0.96	9.30 ± 1.20 ^*NC^	14.72 ± 1.74 ^*NC^	17.96 ± 1.90
KAN	4.53 ± 0.65	9.33 ± 1.33 ^*NC^	14.61 ± 1.69 ^*NC^	18.26 ± 2.29 ^*NC^
RES	2.95 ± 0.44	6.18 ± 0.30 ^*PC^	9.77 ± 1.35 ^*PC^	14.65 ± 1.92 ^*PC^
QUE	2.98 ± 0.07	6.03 ± 0.31 ^*PC^	10.59 ± 1.39 ^*PC^	14.95 ± 2.03 ^*PC^
CUR	2.63 ± 0.34	5.99 ± 0.23 ^*PC^	10.88 ± 1.58 ^*PC^	14.91 ± 2.49 ^*PC^

Legend: * *p* < 0.05; ** *p* < 0.01; *** *p* < 0.001; NC—negative control; PC—positive control; PEN—penicillin; GEN—gentamicin; KAN—kanamycin; RES—resveratrol; QUE—quercetin; CUR—curcumin; ^NC^—compared to the negative control; ^PC^—compared to the positive control.

**Table 8:** The effect of selected antibiotics and bioactive molecules on the counts of *E. faecalis* in induced bacteriospermia.

*E. faecalis* Colonies [×10^8^ CFU/mL]	0 h	2 h	4 h	6 h
NC	0.00 ± 0.00	0.00 ± 0.00	0.00 ± 0.00	0.00 ± 0.00
PC	0.93 ± 0.10 ^*NC^	4.46 ± 1.22 ^***NC^	7.30 ± 1.74 ^***NC^	10.68 ± 2.79 ^***NC^
PEN	0.91 ± 0.15 ^*NC^	1.09 ± 0.12 ^*NC, **PC^	2.06 ± 0.87 ^**NC, **PC^	4.66 ± 1.72 ^***NC, **PC^
GEN	0.90 ± 0.12 ^*NC^	0.55 ± 0.23 ^***PC^	0.60 ± 0.09 ^***PC^	0.77 ± 0.12 ^*NC, ***PC^
KAN	0.94 ± 0.11 ^*NC^	0.93 ± 0.19 ^*NC, ***PC^	1.50 ± 0.65 ^*NC, ***PC^	1.90 ± 1.00 ^**NC, ***PC^
RES	0.91 ± 0.10 ^*NC^	3.88 ± 1.30 ^**NC^	6.06 ± 1.65 ^***NC^	8.05 ± 2.22 ^***NC^
QUE	0.95 ± 0.13 ^*NC^	4.03 ± 1.21 ^***NC^	6.59 ± 1.44 ^***NC^	8.15 ± 2.13 ^***NC^
CUR	0.92 ± 0.15 ^*NC^	2.19 ± 0.33 ^**NC, **PC^	3.88 ± 1.11 ^**NC, **PC^	4.91 ± 1.19 ^***NC, **PC^

Legend: * *p* < 0.05; ** *p* < 0.01; *** *p* < 0.001; NC—negative control; PC—positive control; PEN—penicillin; GEN—gentamicin; KAN—kanamycin; RES—resveratrol; QUE—quercetin; CUR—curcumin; ^NC^—compared to the negative control; ^PC^—compared to the positive control. CFU—colony forming units.

**Table 9:** Experimental design.

	NC	PC	Exp. Group 1	Exp. Group 2	Exp. Group 3	Exp. Group 4	Exp. Group 5	Exp. Group 6
*E. faecalis*	-	+	+	+	+	+	+	+
Supplement	-	-	PEN	GEN	KAN	RES	QUE	CUR

Legend: NC—negative control; PC—positive control; PEN—penicillin; GEN—gentamicin; KAN—kanamycin; RES—resveratrol; QUE—quercetin; CUR—curcumin
